# A Review of the Implementation Status of and National Plans on HPV Vaccination in 17 Middle-Income Countries of the WHO Western Pacific Region

**DOI:** 10.3390/vaccines9111355

**Published:** 2021-11-18

**Authors:** Rei Haruyama, Sumiyo Okawa, Hiroki Akaba, Hiromi Obara, Noriko Fujita

**Affiliations:** 1 Bureau of International Health Cooperation, National Center for Global Health and Medicine, Tokyo 162-8655, Japan; h-obara@it.ncgm.go.jp (H.O.); norikof@it.ncgm.go.jp (N.F.); 2 Institute for Global Health Policy Research, National Center for Global Health and Medicine, Tokyo 162-8655, Japan; sokawa@it.ncgm.go.jp; 3 Life Course and Integration/EPI, Department of Immunization, Vaccines and Biologicals, World Health Organization, 1211 Geneva, Switzerland; akabah@who.int

**Keywords:** cervical cancer, HPV vaccination, Western Pacific Region

## Abstract

The World Health Organization’s Western Pacific Region is responsible for one-fourth of the global cervical cancer burden, and nearly 90% of that burden is concentrated in middle-income countries (MICs). Applying a conceptual model of implementation of population-based interventions, we synthesized the current implementation status of human papillomavirus (HPV) vaccination and national plans that form the basis of its implementation in 17 MICs. We gathered information from a range of governmental documents, published studies, and global databases. For all available national cancer-related plans and immunization plans, we examined the description of HPV vaccination. We found that, as of July 2021, only four countries (24%) had a mature HPV vaccination program with a high first-dose coverage; three (18%) had introduced HPV vaccination, but needed further efforts to scale it up, seven (41%) had not been able to introduce it after conducting demonstration projects, and three (18%) did not have any experience in HPV vaccination. In the national plans, most of the countries recognized the importance of HPV vaccination, but only 10 (59%) provided an implementation strategy on how it would be introduced or scaled up. Countries with a mature program were more likely to have their implementation strategy detailed in their national cancer control plan. Successful implementation of HPV vaccination requires overcoming known challenges and having a clear national plan. Positioning HPV vaccination clearly in the overall national cancer control plan may be key to accelerating its nationwide implementation.

## 1. Introduction

The World Health Organization (WHO)’s Western Pacific Region (WPR) comprises 27 countries and 10 areas, home to 953 million women [1,2]. Of the 27, eight are categorized as high-income countries (HICs), six as upper-middle-income countries, and 11 as lower-middle-income countries as per the World Bank classification (status of two countries is unavailable) [3]. In the WPR, an estimated 146,000 women are newly diagnosed with cervical cancer and 75,000 women die from it every year, accounting for nearly one-fourth of the cases worldwide [4]. Among women of reproductive age (15 to 49 years), mortality from cervical cancer now far exceeds that from maternal conditions (estimated 14,000 and 7900 respectively in 2019), representing a major threat to young women, their families, and communities [5]. Notably, nearly 90% of the WPR’s cervical cancer cases and deaths occur in its middle-income countries (MICs) [4].

Cervical cancer is a devastating disease caused by persistent infection with oncogenic human papillomavirus (HPV) [6]. However, the presence of HPV vaccines and screening tests to detect cervical HPV infection or precancerous lesions also makes it one of the most preventable forms of cancer. In 2020, the WHO Member States adopted the Global Strategy to Accelerate the Elimination of Cervical Cancer as a Public Health Problem, which proposes countries to introduce and scale up HPV vaccination, screening, and treatment, and meet the “90-70-90” targets by 2030 (i.e., 90% of girls are fully vaccinated with HPV vaccine by age 15; 70% of women are screened with a high-performance test by age 35 and again by 45; and 90% of women identified with cervical disease receive treatment) to achieve elimination within this century [7]. Modeling studies suggest that, while screening and treatment are needed to reduce the cancer incidence in the interim decades, HPV vaccination is vital for its elimination [8,9]. In the WPR, however, the introduction of HPV vaccination in the routine immunization schedule has been slow in MICs compared to HICs [10]. The estimated regional vaccine coverage remained as low as 6% for the first dose and 4% for the final dose in 2020 [10]. 

In this review, we synthesized the implementation status of HPV vaccination and national plans that form the basis of its implementation in 17 MICs of the WPR. These countries are highly diverse in terms of population size and health system structure, but all are in the midst of socioeconomic development and rapid aging (Table 1) [1,3,11,12,13]. The national or expanded program on immunization (EPI) generally functions well in terms of DTP3 (third dose of diphtheria–tetanus–pertussis) coverage rate, a widely accepted indicator for immunization program performance [14].

## 2. Materials and Methods

### 2.1. Conceptual Framework

**Figure 1:**
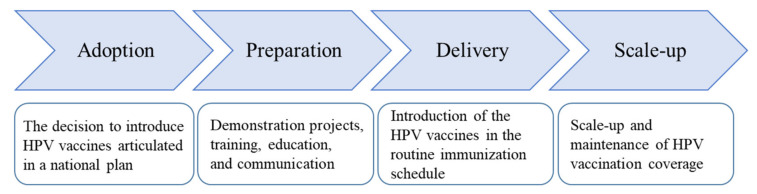
Conceptual model of implementation of population-based interventions and its application to HPV vaccination Adapted from [15,16,17].

To synthesize and present the implementation status of HPV vaccination, we applied a conceptual model of implementation of population-based interventions [15,16,17]. This model proposes four phases of an intervention’s implementation in a public health setting: adoption, preparation, delivery, and scale-up (Figure 1). Adoption involves policy decision-making regarding whether to introduce the intervention based on various factors, including disease burden, cost, the intervention’s affordability and cost-effectiveness, availability of financial and human resources, and compatibility with current processes [15]. Once a decision is made at the national level, it should be articulated in a relevant policy document to facilitate programmatic actions by a host of actors. Preparation entails assessment of readiness, followed by necessary modification in operational structures and processes, as well as training, education, and communication for the intervention’s successful delivery to the target population. Delivery is the actual introduction and dissemination of the intervention, followed by a scale-up to achieve the desired population effect.

Using this model, we first reviewed cervical cancer burden and screening practices as essential background. We then examined, for each country, whether they had conducted any demonstration project for gaining experience in delivering the HPV vaccine prior to nationwide introduction [18,19]. While these projects are not a prerequisite for the introduction of HPV vaccination, we considered them a part of the preparation phase. Next, we reviewed whether the HPV vaccine was introduced in the national immunization schedule, and if so, the related characteristics (e.g., year of introduction, target age, dose schedule, and primary place of delivery) and program coverages from the year of introduction to 2020. Finally, we examined national plans forming the basis of HPV vaccination implementation to identify whether and how HPV vaccination was positioned within a national policy.

### 2.2. Data Sources

We gathered information from a range of documents and databases between May and July 2021.

#### 2.2.1. Cervical Cancer Burden

The estimated cervical cancer incidence and mortality and their estimation method for 2020 were extracted from the GLOBOCAN Cancer Today database [4]. The projection of cervical cancer incidence and mortality in 2040 was obtained from the GLOBOCAN Cancer Tomorrow database [20]. The databases’ Data and Methods section describes in detail the methodology employed for estimation.

#### 2.2.2. Cervical Cancer Screening Practice

To understand the practice of cervical cancer screening, information on the presence and coverage of the national cervical cancer screening program was extracted from the WHO Global Health Observatory [11]. This reflects the official country response to the WHO NCD Country Capacity Survey 2019.

#### 2.2.3. HPV Vaccination Demonstration Projects and National Programs

Information on HPV vaccination demonstration projects and national programs was extracted from governmental documents and published studies. For the latter, PubMed was searched using the keywords “papilloma”, “HPV”, “vaccine”, and “immunization”, and the name of the country. When a discrepancy in information was observed between different sources, the information in governmental documents was prioritized. Documented challenges and barriers to HPV vaccination implementation were also extracted.

In countries with a national program, program coverage of first and final doses for the years after introduction to 2020 were obtained from the WHO/UNICEF HPV Immunization Coverage Estimates [21]. The methodology used for estimation is explained in the Supplementary Materials of a published study [10].

#### 2.2.4. National Plans

Considering the nature of HPV vaccination, two types of multi-year national plans issued by the government (the Ministry of Health [MoH] or its equivalent) were reviewed: cancer-related plan and immunization plan. The former was defined as any document covering strategies to reduce cervical cancer burden, and included a national noncommunicable disease (NCD) plan, national cancer control plan (NCCP), and national cervical cancer control plan, depending on their availability of each country [22]. An immunization plan was defined as a document setting out the direction of the entire national immunization program, such as a comprehensive multi-year plan on immunization [23]. We searched through the WHO NCD Document Repository, International Cancer Control Partnership Portal, and Gavi Portal, as well as government websites, for publicly available plans [24,25,26]. We also used personal contacts to confirm and obtain the most recent national plans—the responses that we received were for China, Philippines, Vietnam, Malaysia, Lao People’s Democratic Republic (Lao PDR), and Mongolia. When multiple versions of the same document were retrieved, only the most recent plan was used. National plans that could not be retrieved from the abovementioned sources were indicated as not available (NA).

### 2.3. Review of the National Plans

For all available plans of each country, we examined whether HPV vaccination was (i) mentioned with an implementation strategy, i.e., time-bound activities and targets for introduction, scale-up, or maintenance; (ii) mentioned without an implementation strategy (e.g., a simple statement such as “HPV vaccination will be implemented” with no information on how it will be achieved); or (iii) not mentioned. Two reviewers (R.H. and S.O.) independently reviewed and rated the plans fitting the abovementioned criteria 2, 1, and 0, respectively. Discrepancies that occurred were discussed within a wider research group to arrive at a consensus.

### 2.4. Data Synthesis

We compiled all data in Microsoft Excel sheets by country. The 17 MICs were initially classified as Asian and Pacific Island and organized by population size. To present the implementation status of HPV vaccination, we then categorized them into the following four groups: scale-up, i.e., those running a national HPV vaccination program for over five years (one cycle of a multi-year national plan in general); delivery, i.e., those who had introduced HPV vaccination (national or subnational) and were in the process of scaling up; preparation, i.e., those who had completed at least one demonstration project, but not yet introduced HPV vaccination; and others.

## 3. Results

### 3.1. Cervical Cancer Burden

Table 2 summarizes the cervical cancer burden for the 17 MICs. The GLOBOCAN estimates for cervical cancer incidence and mortality were available for 12 of them with a female population of over 0.9 million, but not for the smaller Pacific Island countries. In those 12 countries, a total of 127,000 new cases of and 68,000 deaths from cervical cancer were estimated to have occurred in 2020, representing nearly 90% of the cases in the WPR (China alone accounts for 75%). The age-standardized incidence and mortality rates ranged from 6.6 to 29.8 per 100,000 and 3.4 to 20.7 per 100,000 respectively, with a much higher tendency in the Pacific Island countries than in their Asian counterparts; in over half of the 12 countries, these rates were higher than the WPR regional estimates (10.7 and 5.0 respectively). Furthermore, cervical cancer was found to be the second most common cancer among women in seven countries (Philippines, Cambodia, Mongolia, Papua New Guinea, Fiji, Solomon Islands, and Vanuatu).

Future projections show that, if the current situation continues, both incidence and mortality will rise in all the eight countries for which complete data was available. Their total number of deaths is expected to increase by over 30% from 67,992 in 2020 to 90,407 by 2040. 

### 3.2. Cervical Cancer Screening Practice

Among the 17 MICs, 15 (88%) had a national screening program running in 2019 (Table 3). The main testing method used were cytology in 10 countries (59%) and visual inspection with acetic acid (VIA) in five (29%). No country reported the use of HPV-DNA testing at scale. The screening coverage of the target population was less than 50% in all the countries.

### 3.3. Implementation Status of HPV Vaccination

Table 4 presents a summary of the implementation status of HPV vaccination in the 17 MICs as of July 2021 [19,28,29,30,31,32,33,34,35,36,37,38,39,40,41,42,43,44,45,46].

Four countries (Federated States of Micronesia, Marshall Islands, Malaysia, and Fiji) were placed in the scale-up group, having a national HPV vaccination program for over five years. In these countries, nonavalent or bivalent vaccine was provided to girls through an existing school health program on a semi-annual (0, 6 months) basis [28,29,47,48]. Malaysia had continuously achieved over 80% vaccination coverage for both the first and final doses. In the Federated States of Micronesia, Marshall Islands, and Fiji, vaccination coverage had been generally high (>60%) for the first dose, but declined to around 40–60% for the second dose.

Three countries (Philippines, Solomon Islands, and Lao PDR) were placed in the delivery group. In the Philippines, an HPV vaccination program was introduced in 20 priority provinces in 2015 and later expanded to 48 provinces, covering nearly 60% of the total, but not scaled up nationwide, with program coverage remaining relatively low [33,34]. Quadrivalent vaccine was delivered to girls at school on a semi-annual (0, 6 months) basis. In the Solomon Islands and Lao PDR, the vaccine was recently (in 2019 and 2020, respectively) introduced with Gavi’s support following a demonstration project [36,37]. In both countries, quadrivalent vaccine was provided to girls at school on an annual (0, 12 months) basis; therefore, coverage estimates were only available for one or two data points and need to be closely monitored for maintenance.

Among the 10 countries yet to introduce HPV vaccination, seven (China, Vietnam, Cambodia, Mongolia, Papua New Guinea, Vanuatu, and Kiribati) were placed in the preparation group, having completed one or more demonstration projects. The projects’ outcomes were generally reported as good with high vaccination coverage of the target population, barring Mongolia where community resistance was observed during the vaccination campaign (Table S1) [19,32,34,35,36,37,38,39,40,41,42,43,44,45,46]. The remaining three (the island countries of Samoa, Tonga, and Tuvalu) had neither conducted a demonstration project nor introduced HPV vaccination, thus categorized as others (N.B., national rollout began in Tuvalu in September 2021 and the other two countries are preparing national introduction in the coming year, but only limited information was publicly available).

Challenges and barriers to HPV vaccination introduction and scale-up were obtained from nine countries (Table S2) [29,37,44,45,46,49,50]. Their challenges were similar and thus categorized as: (1) difficulty in vaccine delivery (e.g., identification of target girls, reaching out-of-school girls, and cold chain management), (2) low community awareness and resistance, (3) limited coordination between stakeholders (e.g., the education sector, local governments, and civil society), (4) concern about cost and sustainable financing, and (5) insufficient vaccine supply.

### 3.4. National Plans on HPV Vaccination

Of the 17 MICs, cancer-related plans were obtained for 16 (94%) and immunization plans for nine (53%) (Table 5) [28,29,30,34,36,37,48,51,52,53,54,55,56,57,58,59,60,61,62,63,64,65,66,67,68,69,70,71,72,73,74,75,76,77,78]. A total of 35 plans were reviewed, each rated on a scale of 0 to 2 based on the HPV vaccination description. Overall, in 14 countries (82%), HPV vaccination was mentioned in at least one of the plans reviewed (rated 1 or 2 in Table 5). However, only 10 (59%) provided an implementation strategy (i.e., time-bound activities and targets) showing how it would be introduced or scaled up (rated 2).

When we looked at which type of national plan contained an implementation strategy for HPV vaccination in those 10 countries, it differed greatly by country. An interesting relationship was observed between the implementation status and the type of national plan. Among the four countries in the scale-up group, three (Federated States of Micronesia, Marshall Islands, and Malaysia) had described their implementation strategy for HPV vaccination in the NCCP as one of the primary prevention actions to reduce cancer risk; the fourth, Fiji, had a national immunization plan providing actions for achieving HPV vaccine delivery and coverage target, but HPV vaccination was mentioned only in passing in the NCD plan.

In the delivery group, two countries’ (Lao PDR and Solomon Islands) implementation strategies for HPV vaccination were detailed in the national immunization plan as one of the “new vaccines” along with costing and financing projections, but not in the cancer-related plans. It was therefore unclear to what extent the NCD or cancer program was involved in the implementation process.

Three countries in the preparation group (Vietnam, Cambodia, and Mongolia) had described their implementation strategy for HPV vaccination in the national cervical cancer control plan. Their strategies were similar and entailed building political advocacy, raising public awareness, training providers, exploring financing mechanisms, and improving monitoring systems; however, notwithstanding these detailed strategies, there was little to no information in the immunization plan as well as in the broader NCD plan or NCCP. Furthermore, Vietnam’s immunization plan stated that the HPV vaccine would not be introduced within its policy cycle, given the limited government funding and prioritization of other vaccines (measles/rubella and rotavirus) [63]. In Papua New Guinea, the implementation strategy was included in both the immunization and NCD plans. 

Three Pacific Island countries (Samoa, Tonga, and Tuvalu) did not mention HPV vaccination in any of their national plans. As smaller countries might not need disaggregated plans, we further reviewed their overall health strategic plans, but did not find any mention of HPV vaccination.

## 4. Discussion

In this review, we synthesized the implementation status of HPV vaccination and national plans that form the basis of its implementation in 17 MICs in the WPR. As of July 2021, only four countries (24%) had a mature HPV vaccination program with a high first-dose coverage; three (18%) had introduced HPV vaccination, but needed further efforts to scale it up, seven (41%) had not been able to introduce it after conducting demonstration projects, and three (18%) did not have any experience in HPV vaccination. In their national plans, most of the countries recognized the importance of HPV vaccination, but only 10 (59%) provided an implementation strategy on how it would be introduced or scaled up. Countries with a mature program were more likely to have an implementation strategy detailed in their NCCP.

In WPR, as reported globally and from other regions, less-resourced MICs bear the majority of cervical cancer burden [79]. Recognizing the high burden, most of these countries were running a national cervical cancer screening program using cytology or VIA. However, in 15 of the 17 MICs having such a program, the current reported coverage was below 50%. While screening is an effective tool for early detection of precancerous lesions and invasive cancer, to be effective at the population level, it must cover a large proportion of the target population, be conducted with tests of assured accuracy, and include timely access to diagnostic evaluation and treatment of detected cases [17]. In resource-constrained settings, these requirements are often difficult to meet owing to the complex health system challenges. This difficulty in expanding a screening program with assured quality underscores the importance of successful implementation of HPV vaccination.

To synthesize and present the current implementation status of HPV vaccination, we applied a conceptual model of implementation of population-based interventions. This was because we found that a number of studies on these countries described experiences of demonstration projects and national program introduction, but the information was often scattered in isolation, making it difficult to understand where each country currently stands in the implementation process—this includes whether demonstration projects have been conducted, when vaccination was introduced, and how the coverage has changed over time. Categorizing the countries into four groups showed that only four (24%) had a mature HPV vaccination program (scale-up group), three (18%) had introduced HPV vaccination, but needed further efforts to scale it up (delivery group), seven (41%) had not been able to introduce it after conducting demonstration projects (preparation group), and three (18%) did not have any experience in HPV vaccination (as of July 2021). Not only introduction but maintaining high vaccination coverage is important to ensure target girls are well protected and unvaccinated adolescents and young adults also benefit from herd immunity.

The documented challenges to implementation were five-fold: (1) difficulty in vaccine delivery, (2) low community awareness and resistance, (3) limited coordination between stakeholders, (4) concern about cost and sustainable financing, and (5) insufficient vaccine supply. These challenges are consistent with those reported in previous studies, and none have been identified as unique to the WPR [19,80,81,82,83,84].

The first three challenges (vaccine delivery, community awareness, and coordination) can be explained by two unique features of the HPV vaccine that make it different from traditional childhood vaccines [18,84]: first, its optimal age cohort is adolescent girls aged 9–14, which often falls outside the conventional cohort of the routine immunization schedule (infants aged 0–12 months); second, the link between sexual transmission of a virus, potential development of cervical cancer (decades after infection), and the benefit of HPV vaccination may not be easily understood by girls, their parents, and the community. A recent study showed that there was no correlation between DTP3 and HPV1 coverage estimates [10], indicating that an EPI with high performance in conventional vaccine delivery alone may not always be successful in HPV vaccine delivery. The integration of HPV vaccination with other adolescent health interventions has been highlighted as a key facilitator [85,86]. In countries where COVID-19 vaccination for adolescents is going forward, this opportunity and infrastructure could be utilized for HPV vaccination. Among the 17 MICs in our review, Malaysia appears to have been the most successful in addressing these challenges and attaining high vaccination coverage. It has integrated HPV vaccination into the school health service package, a set of health services (e.g., health education, vision screening, body mass index monitoring, and booster doses of measles/rubella and diphtheria/tetanus) provided to children from preschool to age 16 and managed by the Family Health Development Division of the MoH [47]. To raise awareness and ensure good acceptance, in addition to social communication through media, they have actively provided training and support to frontline school health teams and teachers, who are known to be the key influencers of students’ and parents’ perspectives and behavior, and set up dedicated hotlines for the general public to raise concerns [47,87]. Malaysia’s success has been attributed to strong political will as well as collaboration between health and education authorities at national, state, district, and operational levels and a wide range of stakeholders (media, pharmaceutical companies, religious authorities, village heads, etc.) [47,87]. The authors of the cited studies further stress the importance of national policies and operational mechanisms for ensuring such collaboration.

Issues regarding cost and financing are other common challenges that MICs face [88]. The price per vaccine dose varies greatly among the studied countries, depending on whether the country is currently eligible for Gavi support ($4.6), has transitioned out from Gavi support but is eligible to purchase at the commitment price set by the vaccine manufacturers (i.e., prices similar to those offered to Gavi-supported countries), or is required to procure the vaccine on its own [88,89]. As of July 2021, four countries (Cambodia, Lao PDR, Papua New Guinea, and Solomon Islands) are Gavi-supported and two (Vietnam and Kiribati) are eligible to purchase at the commitment price [89]. Yet, only Lao PDR and the Solomon Islands have introduced the vaccine to date. The validity of the current commitment price depends on the type of vaccine and year of transition to the full self-financing phase, but can end as early as 2025 in some countries [89]. A cost-effectiveness study from Vietnam reported that HPV vaccination will be economically attractive only if the country can procure the vaccine at the Gavi price [90]. An increase in the unit price will inventively hinder introduction and scale-up. A majority of the studied MICs (11 countries) are required to procure the vaccine themselves. The median price per dose for self-procuring MICs is reported to be $9.8, which is twice the Gavi price and much higher than those of many other childhood vaccines, which cost less than $0.5 [91]. In addition to the vaccine cost, the cost of delivery poses an additional financial challenge. The most cost-effective and sustainable delivery strategy (school-, facility- or community-based, campaign or non-campaign style) likely differs by country, depending on, for example, the availability of existing school health programs into which HPV vaccination can be easily integrated [80,81]. Therefore, countries seeking to introduce an HPV vaccination program will need to find a delivery strategy that fits their respective health system structure, without being bound by the school-based approach. The fact that it takes decades for the benefit of HPV vaccination to be visible at the population level makes sustainability a critical factor that should be considered at the outset.

Insufficient vaccine supply remains a global concern, including in the 17 MICs [88,92]. Recently, a new bivalent HPV vaccine manufactured in China (Cecolin^®^, Xiamen Innovax Biotech Co., Ltd., Xiamen, China) has obtained the WHO prequalification [92]. This allows the vaccine to enter the global market, and vaccine shortage may ease to some extent. However, whether or not implementation proceeds in other MICs will, in the end, largely depend on the overall cost and availability of funding. Once the vaccine shortage improves and price drops, gender-neutral and older-age group vaccination may possibly become a policy option in MICs where vaccination for girls has been scaled up. Gender-neutral vaccination is reported to be effective in eliminating oncogenic HPV in a high-income setting [93].

To overcome these challenges, particularly with regards to multisectoral collaboration and financing, high-level commitment and clear policy are vital [94,95,96]. To understand how HPV vaccination is positioned in each country’s national policy, we reviewed national cancer-related plans and immunization plans, although there may be more related policy documents such as those on adolescent health. We found that HPV vaccination was mentioned in at least one of the plans of most of the 17 MICs, but only 10 provided an implementation strategy for its introduction and scale-up. Of these, some provided the strategy in the immunization plan, but not in the cancer-related ones, and therefore, the integration with cervical cancer and broader NCD/cancer control efforts was not clear. In some, the strategy was detailed in the cervical cancer control plan, but vaccination had not been introduced. Interestingly, those in the scale-up group were more likely to have their implementation strategy in the NCCP. This may suggest that to scale up HPV vaccination, at least in the future, it should not only be part of the immunization plan or stand-alone cervical cancer control plan, but also be made an integral part of the broader cancer control agenda and deployed within the strategic NCCP.

Because the primary objective of HPV vaccination is to reduce cervical cancer cases, it is essential that countries monitor its incidence trend alongside vaccination coverage. To monitor incidence, a functional national or regional population-based cancer registry (PBCR) is needed [97]. However, nine of the 17 MICs (Cambodia, Lao PDR, Papua New Guinea, Fiji, Solomon Islands, Samoa, Kiribati, Tonga, and Tuvalu) currently do not have one in place [4]. For countries without PBCR, GLOBOCAN estimates were calculated using data from neighboring countries [4]; for example, for Cambodia and Lao PDR, the incidence rates were calculated using data from Thailand and Vietnam. While these estimates are valuable for understanding the disease burden and setting priorities at the national level, they cannot be used to track changes over time or measure the impact of interventions [4,97]. While the current non-availability of a PBCR should not be a reason to delay the introduction of HPV vaccination, it is an indispensable part of cervical cancer control.

The limitations of this review should be noted. First, we did not examine whether national plans were actually implemented as planned, but rather aimed to provide a snapshot of the current situation and the national plans in place. We did our utmost to gather the most updated national plans of the 17 MICs, but there were a few outdated documents and there may be some that we could not obtain. There were also national plans that were mentioned to exist in a document, but were invalid or did not actually exist when we inquired with the MoH of those countries. Second, there may be a case where HPV vaccination has been implemented at the subnational level without it being mentioned in a national plan. Finally, we only studied MICs in the WPR. It would therefore be interesting to carry out a similar analysis of MICs in other countries and regions.

## 5. Conclusions

Although HPV vaccination is vital for the elimination of cervical cancer, only four of the 17 MICs in the WPR have a mature HPV vaccination program; the other 13 need accelerated efforts to proceed with implementation. Successful introduction and scale-up of HPV vaccination is only possible by overcoming known challenges and having a clear national plan with a viable implementation strategy. Positioning HPV vaccination clearly in the overall NCCP, as an essential part of cancer prevention, may be key to accelerating its nationwide implementation.

## Figures and Tables

**Table 1:** Selected socioeconomic, health, and immunization indicators of 17 middle-income countries.

		Socioeconomic Indicators				Health Indicators			Immunization Indicators [13]		
		Income Classification (2021) [3]	Total Female Population (2020) [1]	Total Number of Girls Aged 9–14 Years (2020) [1]	Female Life Expectancy at Birth (Years, 2019) [11]	Total Fertility Rate (Births per Women, 2015–2020) [1]	Prevalence of Current Tobacco Use in Women (%, 2018) [11]	Prevalence of HIV in Women Aged 15–49 Years (%, 2020) [12]	Estimated DTP3 Coverage (%, 2019)	Availability of a National System to Monitor AEFI (2019)	% of Immunization Expenditure Financed Using Government Funds (%, 2016–2019)
Asian countries	China	UM	701,076,000	46,907,000	80.5	1.7	2.1	-	99	Yes	100
	Philippines	LM	54,552,000	6,366,000	73.6	2.6	6.5	<0.1	77	Yes	100
	Vietnam	LM	48,740,000	4,064,000	78.1	2.1	-	<0.1	89	Yes	81
	Malaysia	UM	15,735,000	1,426,000	77.1	2.0	1.0	<0.1	98	Yes	-
	Cambodia	LM	8,557,000	969,000	72.8	2.5	5.7	0.2	92	Yes	28
	Lao PDR	LM	3,624,000	448,000	71.0	2.7	14.4	0.1	68	Yes	32
	Mongolia	LM	1,663,000	172,000	72.8	2.9	6.1	<0.1	98	Yes	93
Pacific Island Countries	Papua New Guinea	LM	4,379,000	580,000	67.4	3.6	-	0.4	35	No	11
	Fiji	UM	442,000	49,000	70.3	2.8	11.1	<0.1	99	Yes	89
	Solomon Islands	LM	338,000	47,000	67.9	4.4	20.1	-	94	Yes	44
	Vanuatu	LM	151,000	22,000	68.3	3.8	3.3	-	90	No	48
	Samoa	UM	96,000	13,000	71.9	3.9	16.7	-	58	Yes	-
	Kiribati	LM	61,000	8000	62.8	3.6	34.8	-	97	No	59
	Federated States of Micronesia	LM	57,000	7000	66.1	3.1	-	-	78	Yes	-
	Tonga	UM	53,000	7000	76	3.6	12.1	-	99	Yes	90
	Marshall Islands	UM	-	-	-	-	-	-	79	Yes	2
	Tuvalu	UM	-	-	-	-	31.4	-	92	Yes	-

AEFI: Adverse events following immunization; DTP3: third dose of diphtheria–tetanus–pertussis; Lao PDR: Lao People’s Democratic Republic; LM: Lower middle; UM: Upper middle.

**Table 2:** Estimated cervical cancer incidence and mortality in 2020 and 2040, by country.

		Population-Based Cancer Registry [4,27]	Incidence (2020) [4]					Mortality (2020) [4]					Projection (2040) [20]	
			Estimation Method ^a^	No.	ASR (per 100,000 Women)	% among All Female Cancers	Ranking among Female Cancers	Estimation Method ^b^	Total (No.)	ASR (per 100,000 Women)	% among all Female Cancers	Ranking among Female Cancers	Incidence No.	Mortality No.
Asian Countries	China	Regional	2b	109,741	10.7	5.2	6	2b	59,060	5.3	5.0	7	116,000	74,600
	Philippines	Regional	2b	7897	15.2	9.1	2	3	4052	7.9	8.9	3	12,900	7360
	Vietnam	Regional	2b	4132	6.6	4.9	8	3	2223	3.4	4.6	7	5930	3720
	Malaysia	Regional	2b	1740	10.2	6.8	4	3	991	5.8	7.1	5	2860	1850
	Cambodia	No	9	1135	14.0	11.4	2	3	643	8.3	10.4	3	1790	1090
	Lao PDR	No	9	371	12.0	8.1	4	3	191	6.7	7.1	3	629	354
	Mongolia	National	2a	334	19.7	12.4	2	2a	182	11.6	9.3	3	514	333
Pacific Island Countries	Papua New Guinea	No	9	1077	29.2	15.9	2	3	650	19.1	17.0	2	1750	1100
	Fiji	No	3b	136	29.8	15.2	2	2a	92	20.7	17.9	2		
	Solomon Islands	No	9	65	25.4	18.1	2	3	40	16.4	25.2	2		
	Vanuatu	National	2a	22	17.1	17.9	2	3	19	14.9	27.9	1		
	Samoa	No	9	10	12.4	9.9	7	9	6	7.5	5.6	6		
	Kiribati	No	-	-	-	-	-	-	-	-	-	-		
	Federated States of Micronesia	National	-	-		-	-	-	-	-	-	-		
	Tonga	No	-	-		-	-	-	-	-	-	-		
	Marshall Islands	National	-	-		-	-	-	-	-	-	-		
	Tuvalu	No	-	-	-	-	-	-	-	-	-	-		
Total (27 countries in WPR)				145,747	10.7				74,864	5.0			159,000	97,600
Total (China, Philippines, Viet Nam, Malaysia, Cambodia, Lao PDR, Mongolia, Papua New Guinea) (% among 27 countries in WPR)				126,427 (87%)					67,992 (91%)				142,373 (90%)	90,407 (93%)

ASR: age-standardized rate; Lao PDR: Lao People’s Democratic Republic; WPR: Western Pacific Region of the World Health Organization. ^a^ Methods used to estimate incidence in GLOBOCAN 2020: 1. National (or local with coverage greater than 50%) rates projected to 2020; 2a. Most recent rates from a single registry applied to 2020 population; 2b. Weighted/simple average of the most recent local rates applied to 2020 population; 3a. Estimated from national mortality estimates by modelling, using mortality to incidence ratios derived from country-specific cancer registry data; 3b. Estimated from national mortality estimates by modelling, using mortality to incidence ratios derived from cancer registry data in neighboring countries; 4. All-sites estimates from neighboring countries partitioned using frequency data; 9. No data: the rates are those of neighboring countries or registries in the same area. ^b^ Methods used to estimate mortality in GLOBOCAN 2020: 1. National rates projected to 2020; 2a. Most recent rates from one source applied to 2020 population; 2b. Weighted/simple average of the most recent local rates applied to 2020 population; 3. Estimated from national incidence estimates by modelling, using incidence to mortality ratios derived from cancer registry data in neighboring countries; 9. No data: the rates are those of neighboring countries in the same area.

**Table 3:** Cervical cancer screening practice in 2019, by country.

		Cervical Cancer Screening Program [11]		
		National Program	Main Testing Method	Screening Coverage (%) *
Asian Countries	China	Yes	Cytology	10–50
	Philippines	Yes	VIA	-
	Vietnam	Yes	Cytology	10–50
	Malaysia	Yes	Cytology	10–50
	Cambodia	Yes	VIA	10–50
	Lao PDR	Yes	Cytology	-
	Mongolia	Yes	Cytology	10–50
Pacific Island Countries	Papua New Guinea	No	-	-
	Fiji	Yes	Cytology	10–50
	Solomon Islands	Yes	VIA	10–50
	Vanuatu	Yes	Cytology	10–50
	Samoa	No	-	-
	Kiribati	Yes	Cytology	10–50
	Federated States of Micronesia	Yes	VIA	10–50
	Tonga	Yes	Cytology	<10
	Marshall Islands	Yes	Cytology	10–50
	Tuvalu	Yes	VIA	10–50

Lao PDR: Lao People’s Democratic Republic; VIA: visual inspection with acetic acid. * Official country response to the WHO NCD Country Capacity Survey 2019. Countries were asked to indicate whether they have a national cervical cancer screening program and its coverage of target population (“less than 10%”, “10% to 50%”, “more than 50% but less than 70%”, or “70% or more”).

**Table 4:** Implementation status of HPV vaccination demonstration projects and national programs (as of July 2021), by country.

		Demonstration Project	National Program				Vaccination Coverage of National Program *												
		Project Year	Introduction Year	Target Population	Vaccine Type; Dosing Schedule	Primary Place of Delivery	’08	’09	’10	’11	’12	’13	’14	’15	’16	’17	’18	’19	’20
Scale-up group	Federated States of Micronesia [28]	-	2009	11–12 yo, girls	9v; 0, 6 m	school		NA NA	NA NA	NA NA	NA NA	NA NA	NA NA	NA NA	71 NA	77 66	70 57	70 57	99 37
	Marshall Islands [29]	-	2009	11–12 yo, girls	9v; 0, 6 m	school		NA NA	30 46	NA NA	20 3	11 10	46 14	31 23	88 31	21 21	NA 28	67 24	NA NA
	Malaysia [30]	-	2010	13 yo, girls	2v; 0, 6 m	school			NA NA	83 80	85 84	91 90	85 85	85 84	83 83	96 96	84 83	86 85	88 84
	Fiji [31,32]	2008–09	2013	12–13 yo, girls	2v; 0, 6 m	school	D	D				98 59	92 89	91 67	93 53	90 56	86 48	93 56	NA NA
Delivery group	Philippines [33,34,35]	2011–12; 2014	2015	9–10 yo, girls	4v; 0, 6 m	school				D	D		D	28 24	34 24	27 4	14 1	23 2	23 5
	Solomon Islands [36]	2015–17	2019	9 yo, girls	4v; 0, 12 m	school								D	D	D		67 NA	NA 22
	Lao PDR [37]	2013–16	2020	10 yo, girls	4v; 0, 12 m	school						D	D	D	D				76 NA
Preparation group	China [38,39]	2020–22; 2021–	-	-	-	-													D
	Vietnam [40]	2008–10	-	-	-	-	D	D	D										
	Cambodia [41,42,43]	2009–10; 2010–11; 2016–17	-	-	-	-		D	D	D					D	D			
	Mongolia [19,44]	2012; 2014	-	-	-	-					D		D						
	Papua New Guinea [19,45]	2012; 2017–18	-	-	-	-					D					D	D		
	Vanuatu [46]	2008; 2015–16	-	-	-	-	D							D	D				
	Kiribati [19]	2011–13	-	-	-	-				D	D	D							
Others	Samoa	-	-	-	-	-													
	Tonga	-	-	-	-	-													
	Tuvalu	-	-	-	-	-													

D: year of demonstration projects; m: months; Lao PDR: Lao People’s Democratic Republic; NA: not available; yo: year-old; 2v: bivalent vaccine; 4v: quadrivalent vaccine; 9v: nonavalent vaccine. * Green cells indicate the years after the national introduction and the numbers show the program coverage of first dose (upper row) and final dose (lower row).

**Table 5:** National plans and description of HPV vaccination (as of July 2021), by country.

		Cancer-Related Plan						Immunization Plan	
		NCD Plan		Cancer Control Plan		Cervical Cancer Control Plan			
		Availablity (Year Covered)	Description of HPV Vaccination *	Availablity (Year Covered)	Description of HPV Vaccination^*^	Availablity (Year Covered)	Description of HPV Vaccination *	Availablity (Year Covered)	Description of HPV Vaccination^*^
Scale-up group	Federated States of Micronesia [28,51]	Yes (2019–24)	1	Yes (2019–24)	2	NA	-	NA	-
	Marshall Islands [29,52]	Yes (2013–18)	0	Yes (2017–22)	2	NA	-	NA	-
	Malaysia [30,53]	Yes (2016–25)	0	Yes (2016–20)	2	NA	-	NA	-
	Fiji [48,54]	Yes (2015–19)	1	NA	-	NA	-	Yes (2013–16)	2
Delivery group	Philippines [34,55,56]	Yes (2017–25)	1	Yes (2015–20)	1	NA	-	Yes (2016–21)	1
	Solomon Islands [36,57]	Yes (2019–23)	0	NA	-	NA	-	Yes (2016–20)	2
	Lao PDR [37,58]	Yes (2014–20)	1	NA	-	NA	-	Yes (2019–23)	2
Preparation group	China [59,60]	Yes (2017–25)	0	Yes (2019–22)	1	NA	-	NA	-
	Vietnam [61,62,63]	Yes (2015–25)	1	NA	-	Yes (2016–25)	2	Yes (2016–20)	1
	Cambodia [64,65,66]	Yes (2018–27)	0	NA	-	Yes (2019–23)	2	Yes (2016–20)	1
	Mongolia [67,68,69,70]	Yes (2017–21)	1	Yes (2015–20)	1	Yes (2018–21)	2	Yes (2017–21)	0
	Papua New Guinea [71,72,73]	Yes (2015–20)	2	Yes (2017–21)	1	NA	-	Yes (2016–20)	2
	Vanuatu [74]	Yes (2016–20)	1	NA	-	NA	-	NA	-
	Kiribati [75]	NA	-	NA	-	NA	-	Yes (2019–23)	1
Others	Samoa [76]	Yes (2018–23)	0	NA	-	NA	-	NA	-
	Tonga [77]	Yes (2015–20)	0	NA	-	NA	-	NA	-
	Tuvalu [78]	Yes (2017–21)	0	NA	-	NA	-	NA	-

Lao PDR: Lao People’s Democratic Republic; NA: not available. * Rated based on the following criteria: 2. HPV vaccination mentioned with implementation strategy (i.e., time-bound activities and targets for introduction and scale-up); 1. HPV vaccination mentioned without implementation strategy (e.g., simple statement such as “HPV vaccination will be implemented in 2018”); 0. HPV vaccination not mentioned at all.

## Data Availability

Publicly available datasets were analyzed in this study described in the Methods section.

## Supplementary Materials

The following are available online at https://www.mdpi.com/article/10.3390/vaccines9111355/s1, Table S1: Characteristics of HPV vaccination demonstration projects, by country; Table S2: Documented challenges and barriers to introduction and scale-up.

## References

[1] United Nations, Department of Economic and Social Affairs, Population Division World Population Prospects 2019. https://population.un.org/wpp/Download/Standard/Population/.

[2] World Health Organization Countries. https://www.who.int/countries.

[3] World Bank New Country Classifications by Income Level: 2021–2022. https://blogs.worldbank.org/opendata/new-world-bank-country-classifications-income-level-2021-2022.

[4] Ferlay J.E.M., Lam F., Colombet M., Mery L., Piñeros M., Znaor A., Soerjomataram I., Bray F., Global Cancer Observatory: Cancer Today https://gco.iarc.fr/today.

[5] World Health Organization (2020). Global Health Estimates 2019: Deaths by Cause, Age, Sex, by Country and by Region, 2000–2019. https://www.who.int/data/gho/data/themes/mortality-and-global-health-estimates/ghe-leading-causes-of-death.

[6] Schiffman M., Castle P.E., Jeronimo J., Rodriguez A.C., Wacholder S. (2007). Human papillomavirus and cervical cancer. Lancet.

[7] World Health Organization (2020). Global Strategy to Accelerate the Eliminate Cervical Cancer as a Public Health Problem. https://www.who.int/publications/i/.

[8] Brisson M., Kim J.J., Canfell K., Drolet M., Gingras G., Burger E.A., Martin D., Simms K.T., Bénard É., Boily M.-C. (2020). Impact of HPV vaccination and cervical screening on cervical cancer elimination: A comparative modelling analysis in 78 low-income and lower-middle-income countries. Lancet.

[9] Simms K.T., Steinberg J., Caruana M., Smith M.A., Lew J.-B., Soerjomataram I., Castle P.E., Bray F., Canfell K. (2019). Impact of scaled up human papillomavirus vaccination and cervical screening and the potential for global elimination of cervical cancer in 181 countries, 2020–99: A modelling study. Lancet Oncol..

[10] Bruni L., Saura-Lázaro A., Montoliu A., Brotons M., Alemany L., Diallo M.S., Afsar O.Z., LaMontagne D.S., Mosina L., Contreras M. (2021). HPV vaccination introduction worldwide and WHO and UNICEF estimates of national HPV immunization coverage 2010–2019. Prev. Med..

[11] World Health Organization The Global Health Observatory. https://www.who.int/data/gho.

[12] UNAIDS (2021). HIV Estimates with Uncertainty Bounds 1990–2020. https://www.unaids.org/en/resources/documents/2021/HIV_estimates_with_uncertainty_bounds_1990-present.

[13] World Health Organization WHO Vaccine-Preventable Diseases: Monitoring System. 2020 Global Summary. https://apps.who.int/immunization_monitoring/globalsummary/.

[14] Sodha S.V., Dietz V. (2015). Strengthening routine immunization systems to improve global vaccination coverage. Br. Med. Bull..

[15] Murillo R., Robles C. (2019). Research Needs for Implementing Cancer Prevention and Early Detection in Developing Countries: From Scientists’ to Implementers’ Perspectives. BioMed Res. Int..

[16] Aarons G.A., Hurlburt M., Horwitz S.M. (2010). Advancing a Conceptual Model of Evidence-Based Practice Implementation in Public Service Sectors. Adm. Policy Ment. Health Ment. Health Serv. Res..

[17] World Health Organization (2020). WHO Report on Cancer: Setting Priorities, Investing Wisely and Providing Care for All. https://apps.who.int/iris/handle/10665/330745.

[18] Hanson C.M., Eckert L., Bloem P., Cernuschi T. (2015). Gavi HPV Programs: Application to Implementation. Vaccines.

[19] Gallagher K.E., Howard N., Kabakama S., Mounier-Jack S., Griffiths U.K., Feletto M., Burchett H., Lamontagne D.S., Watson-Jones D. (2017). Lessons learnt from human papillomavirus (HPV) vaccination in 45 low- and middle-income countries. PLoS ONE.

[20] Ferlay J.L.M., Ervik M., Lam F., Colombet M., Mery L., Piñeros M., Znaor A., Soerjomataram I., Bray F., Global Cancer Observatory: Cancer Tomorrow (2020). https://gco.iarc.fr/tomorrow.

[21] United Nations Children’s Fund (2021). WHO/UNICEF Human Papillomavirus (HPV) Immunization Coverage Estimates. https://data.unicef.org/resources/dataset/immunization/.

[22] Romero Y., Trapani D., Johnson S., Tittenbrun Z., Given L., Hohman K., Stevens L., Torode J.S., Boniol M., Ilbawi A.M. (2018). National cancer control plans: A global analysis. Lancet Oncol..

[23] World Health Organization (2014). WHO-UNICEF Guidelines for Comprehensive Multi-Year Planning for Immunization. https://apps.who.int/iris/bitstream/handle/10665/100618/WHO_IVB_14.01_eng.pdf?sequence=1.

[24] World Health Organization Noncommunicable Disease Document Repository. https://extranet.who.int/ncdccs/documents/Db.

[25] International Cancer Control Partnership ICCP Portal—the One-Stopshop Online Resource for Cancer Planners. https://www.iccp-portal.org.

[26] Gavi, the Vaccine Alliance County hub. https://www.gavi.org/programmes-impact/country-hub.

[27] Tervonen H., Foliaki S., Bray F., Roder D. (2017). Cancer epidemiology in the small nations of Pacific Islands. Cancer Epidemiol..

[28] National Comprehensive Cancer Control Program, Department of Health and Social Affairs (2019). Comprehensive Cancer Control Plan 2019–2024.

[29] Ministry of Health and Human Services (2017). National Comprehensive Cancer Control Plan 2017–2022.

[30] Ministry of Health (2016). National Strategic Plan for Cancer Control Programme 2016–2020.

[31] Ministry of Health and Medical Services (2015). Cervical Cancer Screening Policy.

[32] La Vincente S.F., Mielnik D., Jenkins K., Bingwor F., Volavola L., Marshall H., Druavesi P., Russell F.M., Lokuge K., Mulholland E.K. (2015). Implementation of a national school-based Human Papillomavirus (HPV) vaccine campaign in Fiji: Knowledge, vaccine acceptability and information needs of parents. BMC Public Health.

[33] Philippines Department of Health (2015). Guidelines in the Implementation of HPV Vaccination (DM 2015-0316). https://doh.gov.ph/philippine-cancer-control-program.

[34] Philippines, Department of Health (2016). National Immunization Program. Manual of Operations. Booklet 1. https://doh.gov.ph/sites/default/files/publications/NIP-MOP-Booklet%201.pdf.

[35] Jhpiego (2016). Igniting Change in the Landscape of Health Care for Women and Families. https://www.jhpiego.org/wp-content/uploads/2017/03/Jhpiego-40-Greatest-Hits.pdf.

[36] Solomon Islands Comprehensive Multi-Year Plan for Immunization, 2016–2020. https://www.gavi.org/sites/default/files/document/2021/cMYP%20Solomon%20Islands%202016-2020.pdf.

[37] United States Agency for International Development (2018). National Immunization Programme Updated Comprehensive Multi-year Plan 2019–2023.

[38] Xiamen Municipal Health Commission (2020). Notice on the Implementation Plan of Domestic Bivalent HPV Vaccination in Xiamen.

[39] Office of the People’s Government of Ordos City, China (2020). Notice of the Office of the People’s Government of Ordos City on Issuing the Implementation Plan of the Two-Cancer Prevention and Control Project. http://www.ordos.gov.cn/ordosml/ordoszf/202012/t20201228_2827928.html.

[40] Lamontagne D.S., Barge S., Le N.T., Mugisha E., Penny M.E., Gandhi S., Janmohamed A., Kumakech E., Mosqueira N.R., Nguyen N.Q. (2011). Human papillomavirus vaccine delivery strategies that achieved high coverage in low- and middle-income countries. Bull. World Health Organ..

[41] Garon J., Wuddhika I.V., Sreenivasan N., Wannemuehler K., Vutthikol Y., Chhorvann C., Loharikar A. (2019). Community-based household assessment of human papillomavirus (HPV) vaccination coverage and acceptability—HPV vaccine demonstration program, Cambodia—2017. Vaccine.

[42] Ladner J., Besson M.-H., Hampshire R., Tapert L., Chirenje M., Saba J. (2012). Assessment of eight HPV vaccination programs implemented in lowest income countries. BMC Public Health.

[43] Ladner J., Besson M.-H., Rodrigues M., Audureau E., Saba J. (2014). Performance of 21 HPV vaccination programs implemented in low and middle-income countries, 2009–2013. BMC Public Health.

[44] Batmunkh T., von Mollendorf C., Tulgaa K., Surenjav U., Dalmau M.T., Namjil N., Tsedevdamba B., Tsegmed S., Enkhmaa J., Garland S.M. (2019). HPV genoprevalence and HPV knowledge in young women in Mongolia, five years following a pilot 4vHPV vaccination campaign. Papillomavirus Res..

[45] Waramin E.N.P., Toka D., Mola G., Amos L., Stinshoff J.V., Bagita M. (2017). HPV Vaccination Pilot Program in Papua New Guinea. https://psrh.org.nz/wp-content/uploads/2017/09/HPV-Vaccination-pilot-in-PNG-Mary-Bagita-.pdf.

[46] Harry T.J. (2017). Cervical Cancer Care & HPV Vaccination and Screening in Vanuatu. https://psrh.org.nz/wp-content/uploads/2017/09/Cervical-Cancer-Care-HPV-Vaccinaon-and-screening-In-Vanuatu-Margaret-McAdam-and-Tony-Harry.pdf.

[47] Muhamad N.A., Buang S.N., Jaafar S., Jais R., Tan P.S., Mustapha N., Lodz N.A., Aris T., Sulaiman L.H., Murad S. (2018). Achieving high uptake of human papillomavirus vaccination in Malaysia through school-based vaccination programme. BMC Public Health.

[48] Ministry of Health (2013). National Immunisation Policy and Procedure Manual 2013–2016.

[49] Heffelfinger J. (2018). HPV Vaccination in the Western Pacific and South-East Asia Regions: Overview, Challenges and Opportunities. https://www.sabin.org/sites/sabin.org/files/james_heffelfinger.pdf.

[50] Zhao F., Qiao Y. (2019). Cervical cancer prevention in China: A key to cancer control. Lancet.

[51] Government of Federated States of Micronesia (2019). National Strategic Plan of Action for the Prevention and Control of Non-Communicable Diseases in the Federated States of Micronesia 2019–2024.

[52] Ministry of Health (2013). NCD Emergency Response Towards a Healthy RMI Action Plan 2013–2018.

[53] Ministry of Health (2016). Medium Term Strategic Plan to Further Strengthen the NCD Prevention and Control Program in Malaysia (2016–2025).

[54] Ministry of Health and Medical Services (2015). Non-Communicable Diseases Strategic Plan 2015–2019.

[55] Department of Health (2017). Philippine Strategic Plan for the Prevention and Control Of Noncommunicable Diseases 2017–2025.

[56] Philippines Department of Health National Cancer Prevention and Control Action Plan 2015–2020. https://doh.gov.ph/philippine-cancer-control-program.

[57] Ministry of Health and Medical Services (2019). Multi-Sectoral National Non-communicable Disease Strategic Plan 2019–2023.

[58] Ministry of Health (2014). National Multisectoral Action Plan for the Prevention and Control of Noncommunicable Diseases 2014–2020.

[59] National Health Commission (2019). Healthy China Action Cancer Control Implementation Plan 2019–2022.

[60] Office of the State Council (2017). Long-Term Plan for the Prevention and Treatment of Chronic Diseases (2017–2025).

[61] Ministry of Health (2015). National Strategy on Prevention and Control of Cancer, Cardiovascular Diseases, Diabetes, Chronic Obstructive Pulmonary Diseases, Asthma and Other NCDs Period 2015–2025.

[62] Ministry of Health (2016). National Action Plan for Prevention and Control of Cervical Cancer 2016–2025.

[63] Ministry of Health (2016). Comprehensive Multi-Year Plan for Extended Program on Immunization 2016–2020.

[64] Ministry of Health (2016). National Immunization Program Strategic Plan 2016–2020.

[65] Ministry of Health (2018). National Multisectoral Action Plan for the Prevention and Control of Noncommunicable Diseases 2018–2027.

[66] Ministry of Health (2019). National Action Plan for Cervical Cancer Prevention and Control 2019–2023.

[67] Ministry of Health (2015). Sub-Program on Cancer Prevention and Control 2015–2020.

[68] Ministry of Health (2017). National Program on NCD Control.

[69] Ministry of Health (2018). United Nations Joint Global Program to Reduce Cervical Cancer Morbidity and Mortality in Mongolia in 2018–2021.

[70] Ministry of Health (2017). National Infectious Disease Prevention and Control Program 2017–2021.

[71] National Department of Health (2015). National Multisetoral Strategic Plan for the Prevention and Control of NonCommunicable Diseases 2015–2020.

[72] National Department of Health (2016). Comprehensive Epi Multi-Year Plan For National Immunization Programme 2016–2020.

[73] National Department of Health (2017). Cancer Action Priorities for 2017–2021.

[74] Ministry of Health (2016). Non-communicable Disease Policy and Strategic Plan 2016–2020.

[75] Ministry of Health & Medical Services (2019). Comprehensive Multi-Year Plan for Immunization & Nutrition 2019–2023.

[76] Ministry of Health (2018). National Noncommunicable Disease Control Policy 2018–2023.

[77] Ministry of Health (2015). National Strategy for Prevention and Control of Non-Communicable Diseases 2015–2020.

[78] Ministry of Health (2017). National Noncommunicable Diseases Strategic Plan 2017–2021.

[79] Arbyn M., Weiderpass E., Bruni L., de Sanjosé S., Saraiya M., Ferlay J., Bray F. (2020). Estimates of incidence and mortality of cervical cancer in 2018: A worldwide analysis. Lancet Glob. Health.

[80] Gallagher K., LaMontagne D., Watson-Jones D. (2018). Status of HPV vaccine introduction and barriers to country uptake. Vaccine.

[81] Tsu V.D., LaMontagne D.S., Atuhebwe P., Bloem P.N., Ndiaye C. (2021). National implementation of HPV vaccination programs in low-resource countries: Lessons, challenges, and future prospects. Prev. Med..

[82] Kumar S., Khanduri A., Sidibe A., Morgan C., Torode J., Basu P., Bhatla N., Schocken C., Bloem P. (2021). Acting on the call: A framework for action for rapid acceleration of access to the HPV vaccination in low-and lower-middle-income countries. Int. J. Gynecol. Obstet..

[83] Toh Z.Q., Russell F.M., Garland S.M., Mulholland E.K., Patton G., Licciardi P.V. (2021). Human Papillomavirus Vaccination After COVID-19. JNCI Cancer Spectr..

[84] World Health Organization (2016). Guide to Introducing HPV Vaccine into National Immunization Programmes. https://apps.who.int/iris/bitstream/handle/10665/253123/9789241549769-eng.pdf?sequence=1&isAllowed=y.

[85] Hindin M.J., Bloem P., Ferguson J. (2015). Effective Nonvaccine Interventions to Be Considered Alongside Human Papilloma Virus Vaccine Delivery. J. Adolesc. Health.

[86] Broutet N., Lehnertz N., Mehl G., Camacho A.V., Bloem P., Chandra-Mouli V., Ferguson J., Dick B. (2013). Effective Health Interventions for Adolescents That Could Be Integrated with Human Papillomavirus Vaccination Programs. J. Adolesc. Health.

[87] Buang S.N., Ja’Afar S., Pathmanathan I., Saint V. (2018). Human papillomavirus immunisation of adolescent girls: Improving coverage through multisectoral collaboration in Malaysia. BMJ.

[88] World Health Organization (2020). Global Market Study: HPV Vaccines. https://www.who.int/immunization/programmes_systems/procurement/mi4a/platform/module2/HPV_Global_Market_Study_Public_Summary-Nov2020.pdf?ua=1.

[89] World Health Organization (2018). Vaccine Pricing: Gavi Fully Self-financing & Accelerated Transition Countries. https://www.who.int/immunization/programmes_systems/procurement/mi4a/platform/module2/Factsheet_vacc_pricing_Gavi_transitioning.pdf.

[90] Van Minh H., My N.T.T., Jit M. (2017). Cervical cancer treatment costs and cost-effectiveness analysis of human papillomavirus vaccination in Vietnam: A PRIME modeling study. BMC Health Serv. Res..

[91] UNICEF Supply Division (2021). Vaccines Pricing Data. https://www.unicef.org/supply/vaccines-pricing-data.

[92] World Health Organization Prequalification of Medical Products (IVDs, Medicines, Vaccines and Immunization Devices, Vector Control). Cecolin®. https://extranet.who.int/pqweb/content/cecolin%C2%AE.

[93] Vänskä S., Luostarinen T., Baussano I., Apter D., Eriksson T., Natunen K., Nieminen P., Paavonen J., Pimenoff V.N., Pukkala E. (2020). Vaccination with Moderate Coverage Eradicates Oncogenic Human Papillomaviruses If a Gender-Neutral Strategy Is Applied. J. Infect. Dis..

[94] Obel J., McKenzie J., Buenconsejo-Lum L., Durand A., Ekeroma A., Souares Y., Hoy D., Baravilala W., Garland S., Kjaer S. (2015). Mapping HPV Vaccination and Cervical Cancer Screening Practice in the Pacific Region-Strengthening National and Regional Cervical Cancer Prevention. Asian Pac. J. Cancer Prev..

[95] Sankaranarayanan R., Basu P., Kaur P., Bhaskar R., Singh G.B., Denzongpa P., Grover R.K., Sebastian P., Saikia T., Oswal K. (2019). Current status of human papillomavirus vaccination in India’s cervical cancer prevention efforts. Lancet Oncol..

[96] Black E., Richmond R. (2018). Prevention of Cervical Cancer in Sub-Saharan Africa: The Advantages and Challenges of HPV Vaccination. Vaccines.

[97] Piñeros M., Saraiya M., Baussano I., Bonjour M., Chao A., Bray F. (2021). The role and utility of population-based cancer registries in cervical cancer surveillance and control. Prev. Med..

