# Supplementary material for: A Review of the Implementation Status of and National Plans on HPV Vaccination in 17 Middle-Income Countries of the WHO Western Pacific Region
Source: Vaccines (Basel). 2021 Nov 18;9(11):1355. doi: 10.3390/vaccines9111355 (PMC8626009; doi:10.3390/vaccines9111355)
Supplement: Supplementary file 1 [file vaccines-09-01355-s001.zip › vaccines-1420613-supplementary.pdf]

Supplementary material

Table S1. Characteristics of HPV vaccination demonstration projects, by country

Countries	Year (funding)	Target population	Project sites	Delivery strategy	Vaccine type	Dosing schedule	Number of target girls	Program coverage
Fiji [32]	2008-09	9-12 yo, girls	National	School	Quadrivalent	0, 2, 6 m	30,338	55% (3 doses)
Philippines [34, 35]	2011-12 (Jhpigo)	9-13 yo, girls	2 provinces (Cebu, Quezon)	Facility	Quadrivalent	0, 2, 6 m	4000	88% (3 doses)
	2014 (MoH)	-	2 regions (Cordillera, Central Visayas)	Facility	-	-	-	-
Solomon Islands [36]	2015-17 (Gavi)	9-12 yo, girls	2 provinces (Honiara, Isabel)	School + facility + out reach	Quadrivalent	0, 12 m	8,000	-
Lao PDR [37]	2013-16 (Gavi)	10 yo, girls	Capital and 1 province (Vientiane)	School	Quadrivalent	0, 2, 6 m (2013-14); 0, 12 m (2014-16)	-	94-97% (3 doses)
China [38, 39]	2021- (local government)	13-18 yo, girls	1 city (Inner Mongolia)	Facility	Bivalent (Cecolin®)	0, 6 m	11,000	-
	2020-22 (local government)	13-14 yo, girls	1 city (Xiamen)	Facility	Bivalent (Cecolin®)	0, 6 m	-	-
Vietnam [40]	2008-10 (PATH/MoH)	11 yo, girls	3 districts (Quan Hoa, Nong Cong, Ninh Kieu)	School + facility	Quadrivalent	0, 2, 6 m	3,302	96% (3 doses)
		11 yo, girls	3 districts (Quan Hoa, Nong Cong, Binh Thuy)	Facility	Quadrivalent	0, 2, 6 m	2,712	99% (3 doses)
Cambodia [41-43]	2009-10 (GAP)	11-18 yo, girls	1 health facility	Facility	Quadrivalent	0, 2, 6 m	2,000	101%* (3 doses)
	2010-11 (GAP)	-	10 sites	School + facility	Quadrivalent	0, 2, 6 m	9600	78% (3 doses)
	2016-17 (Gavi)	9 yo, girls	2 provinces (Svay Rieng, Siem Riap)	School + facility	Bivalent (Cervarix®)	0, 6 m	11,646	84% (2 doses)
Mongolia [19, 44]	2012 (GAP)	11-17 yo, girls	2 districts in capital (Ulaanbaatar) and 2 provinces (Umungobi, Selenge)	School + facility + out reach	Quadrivalent	0, 2, 6 m	14,063	65% (3 doses)
	2014 (GAP)	-	-	School	-	-	-	-
Papua New Guinea [19, 45]	2012 (GAP)	-	-	School + facility	-	-	-	-
	2017-18 (Rotary)	9-14 yo, girls	Capital (Port Moresby)	School	Quadrivalent	0, 6 m	28,000	-
Vanuatu [46]	2008	-	1 city (North Efate)	-	-	-	-	94% (3 doses)
	2015-16 (Frazer Family Foundation)	10 yo, girls	1 province (Shefa)	School	Bivalent (Cervarix®)	0, 12 m	1000	84% (2 doses)
Kiribati [19]	2011-13 (GAP/ACCF)	-	-	School	-	-	-	-

ACCF: Australian Cervical Cancer Foundation; EPI: Expanded Program on Immunization; GAP: Gardasil® Access Program; m: months; Lao PDR: Lao People's Democratic Republic; MoH: Ministry of Health; yo: year-old

*Suggested to be due to several factors such as errors in census data and attendance of girls near to but not within the target area

Supplementary material

Table S2. Documented challenges and barriers to introduction and scale-up

Countries	Challenges
Marshall Islands [29]	• Limited awareness about the HPV vaccine especially among parents<br>• Limited involvement of women's groups in promoting vaccination<br>• Need for stronger partnership with other health programs
Malaysia [49]	• Reaching out-of-school girls<br>• Linking HPV vaccination data to women's health program<br>• Emerging anti-vaccine groups
Fiji [49]	• Community concerns about vaccine safety<br>• Inadequacy of cold chain<br>• Delays in vaccine delivery and increases in shipping cost<br>• Need for donor assistance to sustain programme
Lao PDR [37]	• Reaching out-of-school girls
China [50]	• Cost of HPV vaccine<br>• Vaccine supplies<br>• Lack of public awareness<br>• Community concern about vaccine safety and efficacy
Cambodia [49]	• Identifying and enumerating the target girls<br>• Limited involvement of province and district education departments<br>• Limited health education given to girls before school-based vaccination
Mongolia [44]	• Community resistance and withdrawal
Papua New Guinea [45]	• Different interests and priorities among stakeholders<br>• Delays in development of operational plan and guidelines<br>• Restrains in funding<br>• Cold chain requirements
Vanuatu [46]	• Vehicle access to schools (tough road and petrol issues)<br>• Tracking of girls for the second dose<br>• Coordination with local government departments and non-governmental organizations

Lao PDR: Lao People's Democratic Republic
